# Relationship between lipoprotein(a) and colorectal cancer among inpatients: a retrospective study

**DOI:** 10.3389/fonc.2023.1181508

**Published:** 2023-05-05

**Authors:** Huijie Wang, Huanwei Zheng, Ping Meng, Xu Cao, Jinli Liu, Teng Zhang, Haiying Zuo, Zhichao Wang

**Affiliations:** ^1^ Department of Endoscopy, Shijiazhuang Traditional Chinese Medicine Hospital, Shijiazhuang, China; ^2^ Department of Gastroenterology, Shijiazhuang Traditional Chinese Medicine Hospital, Shijiazhuang, China; ^3^ Institute of Traditional Chinese Medicine, North China University of Science and Technology, Tangshan, China; ^4^ Graduate School, Hebei North University, Zhangjiakou, China

**Keywords:** lipoprotein(a), colorectal cancer, association, retrospective study, case-control study

## Abstract

The present study was to explore the association between lipoprotein(a) [Lp(a)] and colorectal cancer (CRC) among inpatients. This study included 2822 participants (393 cases vs. 2429 controls) between April 2015 and June 2022. Logistic regression models, smooth curve fitting, and sensitivity analyses were performed to investigate the relationship between Lp(a) and CRC. Compared with the lower Lp(a) quantile 1 (<79.6 mg/L), the adjusted odds ratios (ORs) in quantile 2 (79.6-145.0 mg/L), quantile 3 (146.0-299.0 mg/L), and quantile 4 (≥300.0 mg/L) were 1.41 (95% confidence interval [CI]: 0.95–2.09), 1.54 (95% CI: 1.04–2.27), 1.84 (95% CI: 1.25–2.7), respectively. A linear relationship between lipoprotein(a) and CRC was observed. The finding that Lp(a) has a positive association with CRC supports the “common soil” hypothesis of cardiovascular disease (CVD) and CRC.

## 1 Introduction

CRC is the third most prevalent and second most fatal cancer worldwide, responsible for approximately one in ten cancer cases and deaths in 2020, and is a significant burden on health systems (1). Current evidence supports the so-called “common soil” hypothesis in the pathogenesis of CVD and CRC (2–7), implying that the two conditions share several pathophysiological mechanisms and risk factors. Due to the thrombogenic and atherogenic properties of Lp(a) (8, 9), a prospective cohort study has shown that a high Lp(a) level is a risk factor for CVD. Therefore, we proposed the hypothesis that Lp(a) may be associated with CRC.

Lp(a) is made up of a low-density lipoprotein (LDL) core, which is produced by the liver, and an apolipoprotein B-100 molecule, which is covalently bonded to apolipoprotein(a) [Apo(a)] (10). The Lp(a) level is genetically determined, only slightly influenced by age, gender, and environmental factors, and is stable in healthy (11, 12). Recently, the role of Lp(a) in tumors has attracted increasing attention due to its potential role in tumor angiogenesis, which is a key step in tumor expansion and metastasis (13–18). However, experimental studies have also reported anti-angiogenic and anti-tumor effects (14, 19, 20).

The association between Lp(a) and CVD is well documented (21–25), while the relationship between Lp(a) and cancers (including breast, lung, prostate, colorectal, and liver cancers) has been reported (26–30), but is sparse and controversial. Furthermore, the relationship between lipoprotein(a) and CRC has not been reported in the study with a large sample size.

Given the unclear relationship between Lp(a) and CRC, we conducted the present study based on clinical data from a tertiary hospital in northern China, to explore the association between Lp(a) and CRC.

## 2 Materials and methods

**Figure 1:**
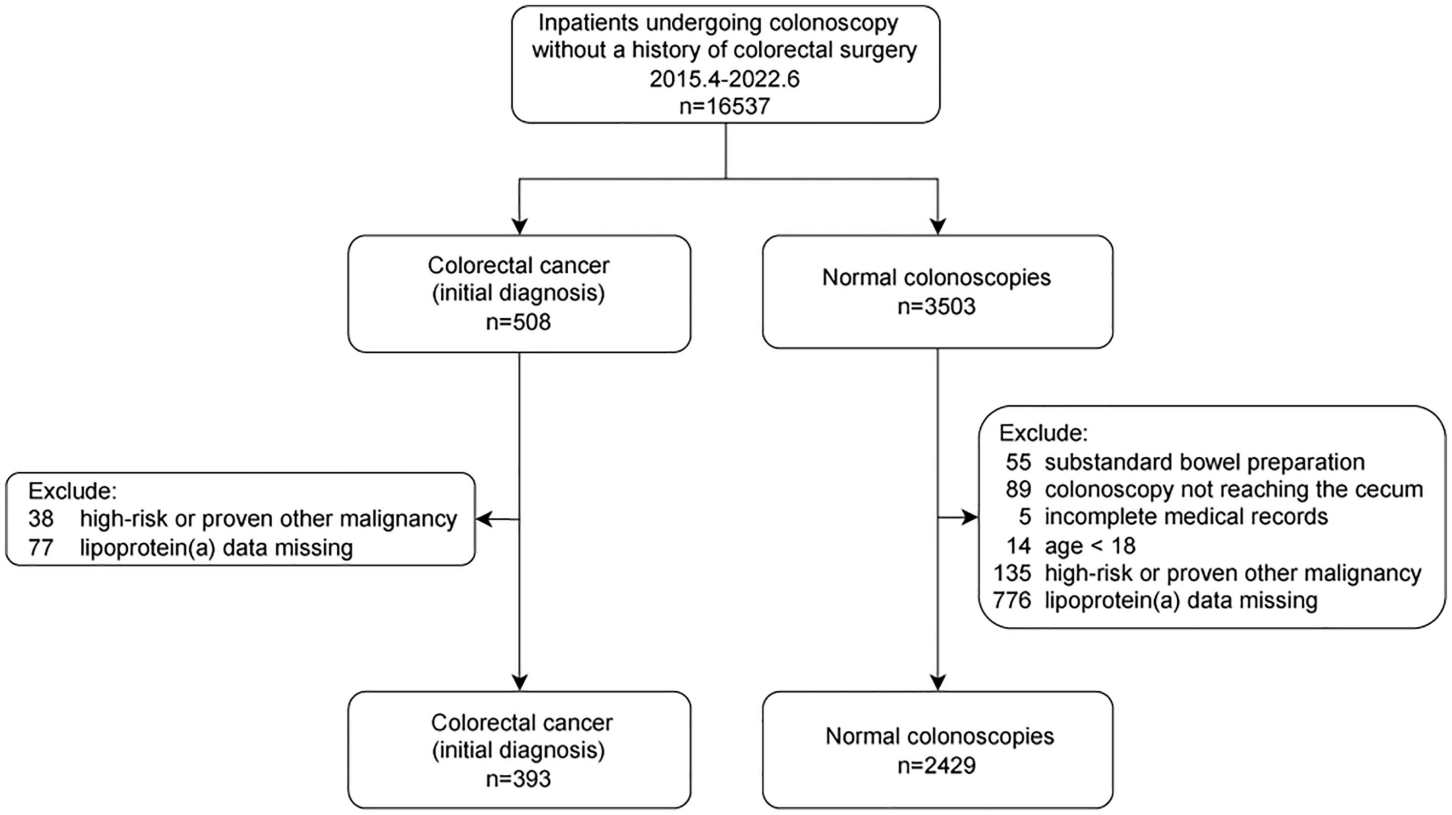
Flowchart of participants.

All consecutive inpatients who underwent colonoscopies were included between April 2015 and June 2022. Each patient was included only once. Subjects with CRC (initial diagnosis) or normal colonoscopy were considered cases or controls, respectively. The detailed inclusion and exclusion criteria are shown in **Figure 1**. Finally, 2822 individuals (393 individuals with CRC vs. 2429 controls) were enrolled.

Several potential covariates were extracted from the laboratory information system (LIS) and hospital information system (HIS) at Shijiazhuang Traditional Chinese Medicine Hospital, including demography, co-morbidities, and laboratory data. Details are shown in **Table 1**. Classification of marital, drinking, and smoking statuses were described in our previous study, as well as liver disease (31). Laboratory indicators were extracted from the first test results during hospitalization.

**Table 1:** Baseline characteristics of participants.

Variables	Total n = 2822	Case n = 393	Control n = 2429	*P* value
Age, year	53.6 ± 13.2	65.6 ± 10.9	51.7 ± 12.5	**< 0.001**
Sex, male, n (%)	1227 (43.5)	238 (60.6)	989 (40.7)	**< 0.001**
Marital status				0.503
Single/divorced	127 (4.5)	20 (5.1)	107 (4.4)	
Married	2542 (90.1)	356 (90.6)	2186 (90)	
Others	153 (5.4)	17 (4.3)	136 (5.6)	
Weight, kg	67.1 ± 12.3	68.1 ± 11.4	67.0 ± 12.4	0.101
Smoking status, n (%)				**0.002**
Non-smoker	1791 (63.5)	265 (67.4)	1526 (62.8)	
Current smoker	103 (3.6)	25 (6.4)	78 (3.2)	
Ex-smoker	23 (0.8)	3 (0.8)	20 (0.8)	
NA	905 (32.1)	100 (25.4)	805 (33.1)	
Drinking status, n (%)				0.091
Non-drinker	1780 (63.1)	267 (67.9)	1513 (62.3)	
Current drinker	132 (4.7)	18 (4.6)	114 (4.7)	
Ex-drinker	15 (0.5)	3 (0.8)	12 (0.5)	
NA	895 (31.7)	105 (26.7)	790 (32.5)	
Family history, n (%)				
Colorectal cancer	24 (0.9)	3 (0.8)	21 (0.9)	1
Digestive system malignancy	117 (4.1)	15 (3.8)	102 (4.2)	0.724
Lp(a), mg/L	146.0 (79.2, 300.0)	198.0 (100.0, 384.0)	138.0 (77.0, 290.0)	**< 0.001**
HDL, mmol/L	1.3 ± 0.3	1.3 ± 0.3	1.4 ± 0.3	**< 0.001**
LDL, mmol/L	2.9 ± 0.7	2.8 ± 0.7	2.9 ± 0.7	0.064
TC, mmol/L	4.9 ± 1.0	4.8 ± 1.0	5.0 ± 1.0	**0.003**
TG, mmol/L	1.3 (0.9, 1.9)	1.3 (0.9, 1.7)	1.3 (0.9, 1.9)	0.521
Apo A1, g/L	1.3 ± 0.3	1.2 ± 0.3	1.3 ± 0.3	**< 0.001**
Apo B, g/L	1.0 ± 0.3	1.0 ± 0.2	1.0 ± 0.3	0.805
TP, g/L	71.5 ± 5.5	69.4 ± 6.0	71.8 ± 5.3	**< 0.001**
ALB, g/L	43.9 ± 3.8	41.9 ± 4.2	44.3 ± 3.7	**< 0.001**
ALT, U/L	17.0 (12.0, 25.0)	14.0 (10.5, 18.0)	17.0 (13.0, 26.0)	**< 0.001**
GGT, U/L	19.0 (14.0, 28.0)	19.0 (15.0, 29.0)	19.0 (14.0, 28.0)	0.227
ALP, U/L	76.9 ± 29.7	88.5 ± 48.3	75.0 ± 25.0	**< 0.001**
AST, U/L	19.0 (16.0, 24.0)	18.0 (15.0, 21.0)	20.0 (16.4, 24.0)	**< 0.001**
ChE, U/L	8774.0 ± 2005.6	7533.2 ± 1878.5	8974.8 ± 1953.0	**< 0.001**
CREA, μmol/L	64.2 ± 20.0	69.7 ± 31.6	63.3 ± 17.3	**< 0.001**
Urea, mmol/L	4.8 ± 1.5	5.1 ± 1.8	4.7 ± 1.5	**< 0.001**
UA, μmol/L	303.0 ± 93.4	303.7 ± 87.8	302.8 ± 94.3	0.862
GLU, mmol/L	6.0 ± 2.0	6.7 ± 2.7	5.9 ± 1.8	**< 0.001**
TBIL, μmol/L	12.6 (9.8, 16.4)	11.8 (8.5, 15.0)	12.7 (10.0, 16.6)	**< 0.001**
DBIL, μmol/L	2.7 (2.0, 3.8)	2.8 (2.0, 3.8)	2.7 (2.0, 3.8)	0.217
β2-MG, mg/L	1.5 (1.3, 1.9)	2.0 (1.6, 2.5)	1.5 (1.2, 1.8)	**< 0.001**
TBA, μmol/L	2.5 (1.4, 4.4)	3.1 (1.6, 5.1)	2.5 (1.4, 4.3)	**< 0.001**
Co-morbidities, n (%)				
Hypertension	730 (25.9)	151 (38.4)	579 (23.8)	**< 0.001**
Ischemic cerebrovascular disease	338 (12.0)	52 (13.2)	286 (11.8)	0.409
CHD	358 (12.7)	51 (13)	307 (12.6)	0.852
HLP	301 (10.7)	18 (4.6)	283 (11.7)	**< 0.001**
Liver disease	326 (11.6)	23 (5.9)	303 (12.5)	**< 0.001**
DM	321 (11.4)	71 (18.1)	250 (10.3)	**< 0.001**

Data are presented mean ± SD, median (quartile 1–quartile 3), or N (%). Bold values indicate statistical significance.

Lp(a), lipoprotein(a); HDL, high-density lipoprotein; LDL, low-density lipoprotein; TC, total cholesterol; TG, triglyceride; Apo A1, apoprotein A1; Apo B, apolipoprotein B; TP, total protein; ALB, albumin; ALT, alanine aminotransferase; GGT, gamma-glutamyl transferase; ALP, alkaline phosphatase; AST, aspartate aminotransferase; ChE, cholinesterase; CREA, creatinine; UA, uric acid; GLU, glucose; TBIL, total bilirubin; DBIL, direct bilirubin; β2-MG, β2-microglobulin; TBA, total bile acid; CHD, coronary heart disease; HLP, hyperlipidemia; DM, diabetes mellitus; NA, not recorded.

Continuous data were presented as mean ± standard deviation or median (Q1–Q3) values. The Mann-Whitney *U* test or Student’s *t* test for continuous variables and the chi-squared test for categorical variables were performed.

The effect of Lp(a) on CRC was investigated using logistic regression models. To further evaluate the impact of Lp(a), the Lp(a) level (mg/L) was divided into quartiles: Q1 (<79.6), Q2 (79.6-145.0), Q3 (146.0-299.0), and Q4 (≥300.0). We constructed three models: (1) crude model; (2) adjusted for gender and age; and (3) adjusted for gender, age, weight, drinking status, smoking status, marital status, family history of CRC, albumin (ALB), alanine aminotransferase (ALT), β2-microglobulin (β2-MG), high-density lipoprotein (HDL), total cholesterol (TC), hypertension, and diabetes mellitus (DM). These potential confounders were selected based on previous studies or a change in effect estimate of more than 10%. Stratified binary logistic regression model and testing for interactions were used to analyze subgroups. Smooth curve fitting and propensity score matching (PSM) were performed to investigate the association between Lp(a) and CRC. Participants were matched for a fully model using a one-to-one nearest neighbor technique with a calliper width of 0.2. Furthermore, sensitivity analysis was conducted using all complete cases. Based on 5 replications and a chained equation approach method in the R mice procedure, multiple imputations were used to minimize bias and maximize statistical power that might occur to account for missing data.

Data analyses were performed with the statistical software packages R 3.3.2 (http://www.R-project.org, The R Foundation) and Free Statistics software version 1.7.1. *P*<0.05 was considered statistically significant (two-tailed).

## 3 Results

### 3.1 Baseline characteristics

The present study enrolled 503 individuals with CRC and 3503 controls. 1189 individuals were excluded owing to incomplete medical records (n=5), substandard bowel preparation (n=55), colonoscopy note reaching the cecum (n=89), missing data (n=853), age<18 (n=14), high-risk or proven other malignancy (n=173). Consequently, 2822 individuals were included in this study (case: control = 393: 2429). The flowchart is shown (**Figure 1**).

The detailed characteristics are available in **Table 1**. Some significant differences were shown in some variables, including age, sex, smoking status, Lp(a), HDL, TC, apolipoprotein A1 (Apo A1), total protein (TP), ALB, ALT, alkaline phosphatase (ALP), aspartate aminotransferase (AST), cholinesterase (ChE), creatinine (CREA), urea, glucose (GLU), total bilirubin (TBIL), direct bilirubin (DBIL), β2-MG, total bile acid (TBA), hypertension, hyperlipidemia (HLP), liver disease, and DM.

### 3.2 Relationship between Lp(a) and CRC

Multivariable logistic regression analyses were performed to assess the associations between Lp(a) and CRC (**Table 2**). When Lp(a) was a continuous variable, in Model 1 adjusted for gender and age, Lp(a) was positively related to CRC (Lp(a) per 100 mg/L, OR: 1.1, 95% CI: 1.05–1.15, *P*<0.001). Even after adjusting for more potential covariates (Model 2-3), the association remained stable (Lp(a) per 100 mg/L; Model 2: OR, 1.11 95% CI, 1.06–1.16, *P*<0.001; Model 3: OR, 1.08, 95% CI, 1.03–1.13, *P*=0.002). Overall, in all models (Crude model and Model 1-3), the risk of advanced colorectal adenomas increased as the level of Lp(a) increased.

**Table 2:** Multivariable logistic regression models of lipoprotein(a) and colorectal cancer.

Variable	Event, n	Crude model		Model I		Model II		Model III	
		OR (95%CI)	*P* value	OR (95%CI)	*P* value	OR (95%CI)	*P* value	OR (95%CI)	*P* value
LP(a), per 100 mg/L	393/2822	1.12 (1.07~1.16)	<0.001	1.1 (1.05~1.15)	<0.001	1.11 (1.06~1.16)	<0.001	1.08 (1.03~1.13)	0.002
LP(a) quartile, mg/L									
Q1 (<79.6)	64/706	1(Reference)		1(Reference)		1(Reference)		1(Reference)	
Q2 (79.6-145.0)	88/704	1.43 (1.02~2.01)	0.038	1.42 (0.98~2.08)	0.066	1.48 (1.01~2.17)	0.044	1.41 (0.95~2.09)	0.09
Q3 (146.0-299.0)	108/699	1.83 (1.32~2.55)	<0.001	1.6 (1.11~2.31)	0.012	1.66 (1.14~2.41)	0.008	1.54 (1.04~2.27)	0.025
Q4 (≥300.0)	133/713	2.3 (1.67~3.16)	<0.001	2.09 (1.46~2.99)	<0.001	2.19 (1.52~3.15)	<0.001	1.84 (1.25~2.7)	0.002
*P* for trend			<0.001		<0.001		<0.001		0.002

Q, quartile; OR, odds ratio; CI, confidence interval; Lp(a), lipoprotein(a); ALB, albumin; ALT, alanine aminotransferase; β2-MG, β2-microglobulin; HDL, high-density lipoprotein; TC, total cholesterol; DM, diabetes mellitus; CRC, colorectal cancer.

Crude model: no other covariates were adjusted.

Model I: adjusted for sex and age.

Model II: adjusted for Model I + weight, marital status, family history of CRC, drinking status, and smoking status.

Model III: adjusted for Model II + ALB, ALT, β2-MG, HDL, TC, hypertension, and DM.

**Figure 2:**
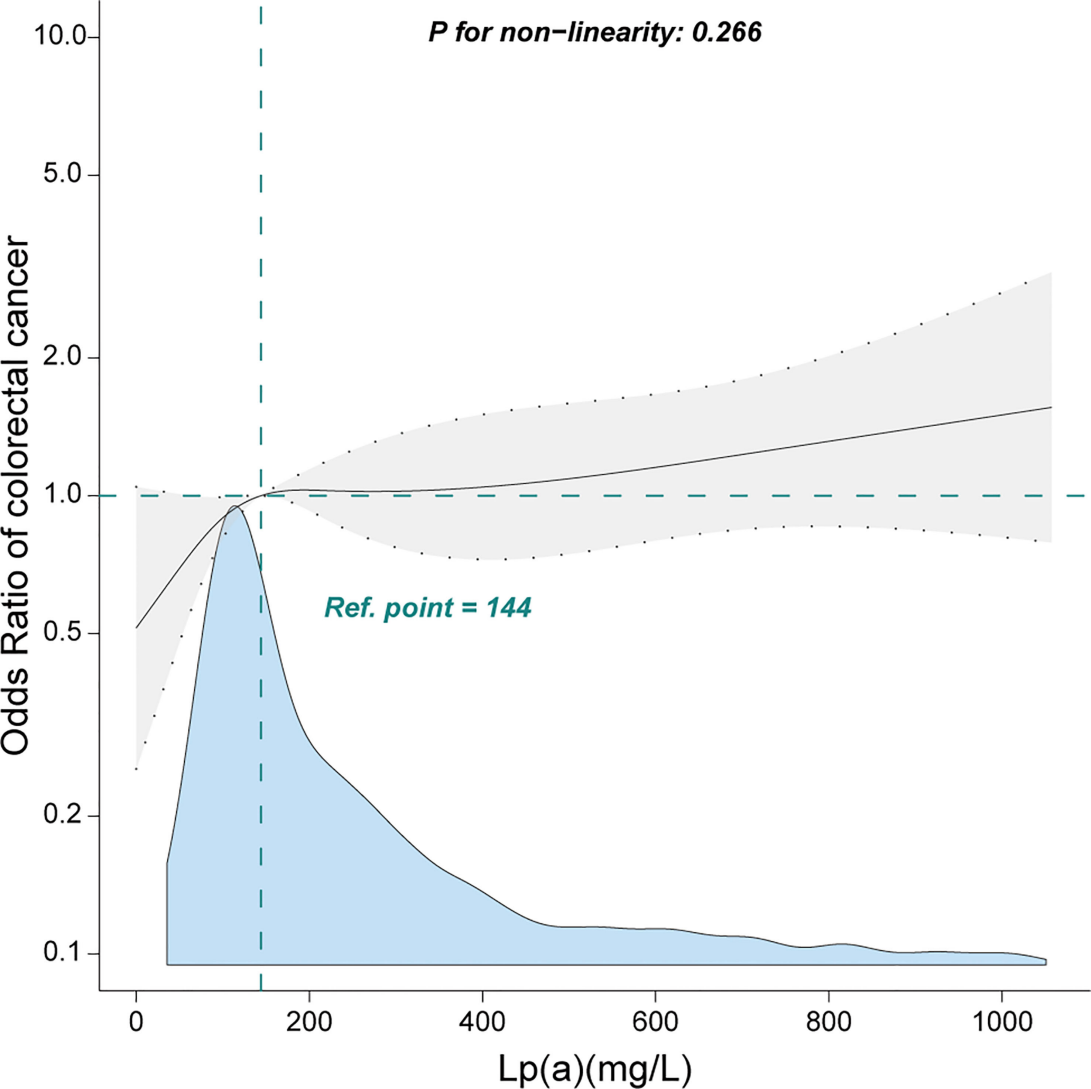
Linear relationship between lipoprotein(a) and colorectal cancer among inpatients. Adjustment factors included gender, age, weight, marital status, drinking status, smoking status, family history of CRC, albumin, alanine aminotransferase, β2-microglobulin, high-density lipoprotein, total cholesterol, hypertension, and diabetes mellitus. Only 99% of the data is shown.

When Lp(a) was analyzed in terms of quartiles, there was a positive association between Lp(a) and CRC. Compared with the lower Lp(a) Q1 (<79.6 mg/L), the adjusted ORs in Q2 (79.6-145.0 mg/L), Q3 (146.0-299.0 mg/L), and Q4 (≥300.0 mg/L) were 1.41 (95% CI: 0.95–2.09), 1.54 (95% CI: 1.04–2.27), 1.84 (95% CI: 1.25–2.7), respectively (**Table 2**). Furthermore, we observed a linear relationship between Lp(a) and CRC among inpatients after adjusting for several covariates (**Figure 2**, only 99% of the data is shown).

### 3.3 Sensitivity analysis

**Figure 3:**
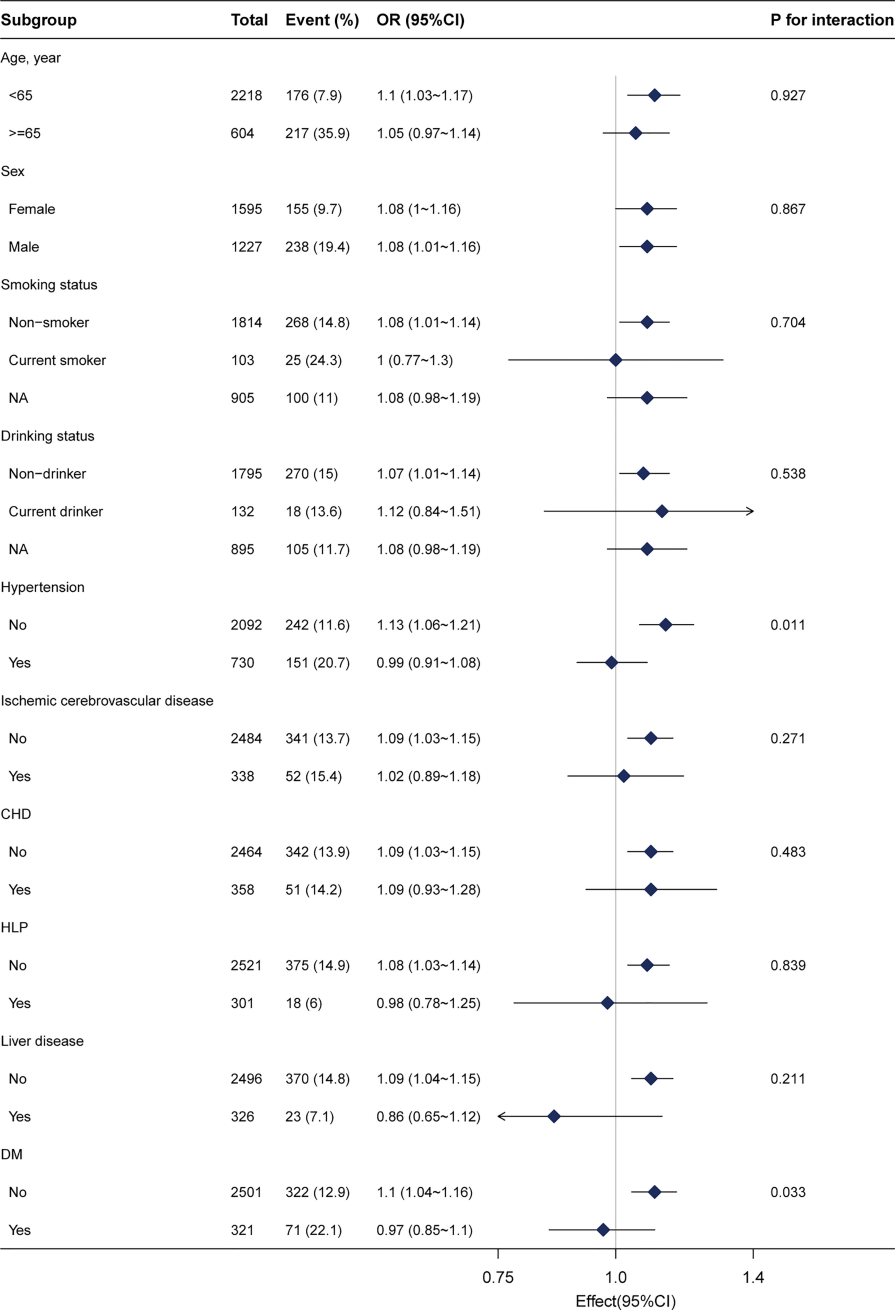
Subgroup analysis of the lipoprotein(a) and colorectal cancer among inpatients. Each stratification factor was adjusted for gender, age, weight, marital status, drinking status, smoking status, family history of CRC, albumin, alanine aminotransferase, β2-microglobulin, high-density lipoprotein, total cholesterol, hypertension, and diabetes mellitus. CHD, coronary heart disease; HLP, hyperlipidemia; DM, diabetes mellitus; NA, not recorded.

Stratified analyses were conducted to investigate potential effects on the association between Lp(a) and CRC. No significant interactions were presented after stratifying by sex, age (<65 years and ≥65 years), drinking status, ischemic cerebrovascular disease, hypertension, liver disease, HLP, coronary heart disease (CHD), and DM (**Figure 3**). Given multiple testing, the *P* value of < 0.05 for the interaction in hypertension and DM subgroups may not be statistically significant. There remained 2604 participants after excluding those with missing data, and the relationship between Lp(a) and CRC remained stable in the sensitivity analysis (**Supplementary Table 1**). Furthermore, propensity score matching analysis was performed in this study (**Supplementary Tables 2, 3**) and indicated the relationship between Lp(a) and CRC remained stable.

## 4 Discussion

This study demonstrated the positive relationship between Lp(a) and CRC. Both subgroup and sensitivity analyses indicated that the relationship remained robust.

There is no doubt that lipid parameters are related to CVD (32–34). Recently, the relationship between Lp(a) and tumors has received much attention. Current studies have shown that Lp(a) was related to certain tumors. Several studies have reported higher levels of Apo(a) or Lp(a) in patients with breast, lung, and prostate cancers [21-23]. While patients with liver cancer had relatively low levels of Lp(a) [24], the reason considered is that the liver being the main site of Apo(a) synthesis, liver cancer affects the expression of Apo(a) protein and consequently the synthesis of Lp(a).

Only one prospective study investigated the relationship between Lp(a) and CRC (n=58), it appeared that the highest levels of Lp(a) had the highest risk of CRC, although there was no significant difference; however, this could not be interpreted as a linear or quadratic relationship (28). In contrast, this present study, which included a larger sample size and adjusted for more covariates, found a linear relationship between Lp(a) and CRC. This may be because they analyzed all cancer sites, in which the effect of type-specific or site-specific cancers may be diluted. To our knowledge, this study is the first study with a larger sample size that found a positive association of Lp(a) with CRC.

The potential mechanisms underlying the association between high Lp(a) and CRC are unclear and require further study. One explanation is that high Lp(a) is more likely to induce the formation of fibrin networks and thrombi, promoting cancer cell adhesion, invasion, and metastasis. This is due to the structural similarity of Lp(a) to fibrinogen and tissue fibrinogen activator and the fact that it competes with fibrinogen for its binding site, leading to reduced fibrinolysis. Another explanation is quite different, as some animal studies have found that the proteolytic breakdown products of Lp(a) have anti-tumor properties both *in vivo* and *in vitro* (27, 29, 30). Considering the anti-tumor effects of Lp(a), we suggest that a high Lp(a) level may be a compensatory response to systemic chronic inflammation caused by aggressive and invasive tumors. Insight into the role of Lp(a) in cancer may shed light on strategies to prevent metastasis (27).

Our findings contribute further data to the common risk factors of CVD and CRC. Studies have shown that lowering Lp(a) levels reduces cardiovascular risk (35–37). We speculate that lowering Lp(a) may reduce the risk of CRC. Current research suggests that several therapies that may reduce cardiovascular risks, such as lifestyle modifications, aspirin, statins, fibrates, and ezetimibe drugs, have little effect on Lp(a) (38, 39). Recent studies have found that the therapeutic agents available to reduce Lp(a) levels are lipoprotein apheresis, small interfering RNA agents, antisense oligonucleotides, and PCSK9 inhibitors (35–37, 40, 41). However, more effects and cardiovascular benefits need to be further investigated.

The study had some limitations. First, missing data is common in observational studies. However, sensitivity analyses indicated that the results were stable. Second, repeated measurements of Lp(a) were lacking in this study and may not be representative of the relationship between long-term levels of Lp(a) and CRC. Third, confounding by unknown or unmeasured factors cannot be completely ruled out despite logistic regression and sensitivity analyses. Finally, these findings were based on clinical data from a tertiary hospital in northern China and require a multi-center study.

## 5 Conclusion

There was a positive relationship between Lp(a) and CRC among inpatients in China. This finding contributes further data to the common risk factors of CVD and CRC and may provide new ideas for the screening or diagnosis of CRC.

## Data availability statement

The raw data supporting the conclusions of this article will be made available by the authors, without undue reservation.

## Ethics statement

The studies involving human participants were reviewed and approved by Ethics Review Board of Shijiazhuang Traditional Chinese Medicine Hospital. Written informed consent for participation was not required for this study in accordance with the national legislation and the institutional requirements.

## Author contributions

HW and HuZ conceived and designed the study; HW, XC, PM, ZW, TZ, and HaZ collected and analyzed the data; HW and HuZ wrote and revised the manuscript. All authors contributed to the article and approved the submitted version.
