# Supplementary material for: Relationship between lipoprotein(a) and colorectal cancer among inpatients: a retrospective study
Source: Front Oncol. 2023 May 5;13:1181508. doi: 10.3389/fonc.2023.1181508 (PMC10196502; doi:10.3389/fonc.2023.1181508)
Supplement: Supplementary file 1 [file Table_1.docx]

**Table S1** Multivariable logistic regression models of lipoprotein(a) and colorectal cancer (n=2604).

| **Variable** | **Event, n** | **Crude model** | |  | **Model I** | |  | **Model II** | |  | **Model III** | |
| --- | --- | --- | --- | --- | --- | --- | --- | --- | --- | --- | --- | --- |
|  |  | **OR (95%CI)** | ***P* value** |  | **OR (95%CI)** | ***P* value** |  | **OR (95%CI)** | ***P* value** |  | **OR (95%CI)** | ***P* value** |
| LP(a), *P*er 100 mg/L | 362/2604 | 1.12 (1.07~1.17) | <0.001 |  | 1.1 (1.05~1.16) | <0.001 |  | 1.11 (1.06~1.17) | <0.001 |  | 1.09 (1.03~1.14) | 0.002 |
| LP(a), quartile, mg/L | |  |  |  |  |  |  |  |  |  |  |  |
| Q1 (<79.6) | 58/651 | 1(Reference) |  |  | 1(Reference) |  |  | 1(Reference) |  |  | 1(Reference) |  |
| Q2 (79.6-144.6) | 78/651 | 1.39 (0.97~1.99) | 0.071 |  | 1.35 (0.91~2.01) | 0.141 |  | 1.38 (0.93~2.06) | 0.114 |  | 1.34 (0.88~2.03) | 0.173 |
| Q3 (144.7-299.0) | 101/639 | 1.92 (1.36~2.71) | <0.001 |  | 1.72 (1.17~2.53) | 0.006 |  | 1.75 (1.18~2.59) | 0.005 |  | 1.65 (1.1~2.48) | 0.016 |
| Q4 (≥300.0) | 125/663 | 2.38 (1.7~3.31) | <0.001 |  | 2.14 (1.47~3.11) | <0.001 |  | 2.24 (1.53~3.27) | <0.001 |  | 1.91 (1.27~2.86) | 0.002 |
| *P* for trend |  |  | <0.001 |  |  | <0.001 |  |  | <0.001 |  |  | 0.001 |

Abbreviations: Q, quartile; OR, odds ratio; CI, confidence interval; LP(a), lipoprotein(a); ALB, albumin; ALT, alanine aminotransferase; Beta2-MG, β2-microglobulin; HDL, high-density lipoprotein; TC, total cholesterol; DM, diabetes mellitus; CRC, colorectal cancer.

Crude model: no other covariates were adjusted.

Model I: adjusted for sex and age.

Model II: adjusted for Model I + weight, marital status, smoking status, drinking status, family history of CRC.

Model III: adjusted for Model II + ALB, ALT, Beta2-MG, HDL, TC, hypertension, and DM.
