# Supplementary material for: Relationship between lipoprotein(a) and colorectal cancer among inpatients: a retrospective study
Source: Front Oncol. 2023 May 5;13:1181508. doi: 10.3389/fonc.2023.1181508 (PMC10196502; doi:10.3389/fonc.2023.1181508)
Supplement: Supplementary file 2 [file Table_2.docx]

Table S2 Association between Lp(a) (per 100 mg/L) and colorectal cancer in the crude analysis, multivariable analysis, and propensity score matching analysis.

| Analysis | OR (95% CI) | *P*-value |
| --- | --- | --- |
| Crude analysis^a^ | 1.12 (1.07~1.17) | <0.001 |
| Multivariable adjusted analysis^b^ | 1.08 (1.03~1.13) | 0.002 |
| Propensity score matched^c^ | 1.07 (1.01~1.14) | 0.027 |

Abbreviations: CI, confidence interval; OR, odds ratio; ALB, albumin; ALT, alanine aminotransferase; β2-MG, β2-microglobulin; HDL, high-density lipoprotein; TC, total cholesterol; DM, diabetes mellitus; CRC, colorectal cancer.

^a^Shown is the OR from the logistic regression model without adjusting any covariates.

^b^Shown is the OR from the multivariable logistic regression model adjusted for Model III (sex, age, weight, marital status, family history of CRC, drinking status, smoking status, ALB, ALT, β2-MG, HDL, TC, hypertension, and DM).

^c^Shown is the OR from the logistic regression model after propensity score matching (matched for Model III). The analysis included 670 participants (335 CRCs and 335 controls).
