# Supplementary material for: Relationship between lipoprotein(a) and colorectal cancer among inpatients: a retrospective study
Source: Front Oncol. 2023 May 5;13:1181508. doi: 10.3389/fonc.2023.1181508 (PMC10196502; doi:10.3389/fonc.2023.1181508)
Supplement: Supplementary file 3 [file Table_3.docx]

Table S3 Baseline characteristics of participants.

| Variables | Original Participants | | |  | Matched Participants | | |
| --- | --- | --- | --- | --- | --- | --- | --- |
|  | Case  n=393 | Control  n=2429 | SMD |  | Case  n=335 | Control  n=335 | SMD |
| Sex, male, n (%) | 238 (60.6) | 989 (40.7) | 0.405 |  | 198 (59.1) | 186 (55.5) | 0.072 |
| Age, year | 65.55 ± 10.91 | 51.68 ± 12.45 | 1.185 |  | 63.46 ± 10.01 | 63.67 ± 9.89 | 0.021 |
| Marital status |  |  | 0.066 |  |  |  | 0.02 |
| Single/ divorced | 20 (5.1) | 107 (4.4) |  |  | 13 (3.9) | 14 (4.2) |  |
| Married | 356 (90.6) | 2186 (90.0) |  |  | 306 (91.3) | 306 (91.3) |  |
| Others | 17 (4.3) | 136 (5.6) |  |  | 16 (4.8) | 15 (4.5) |  |
| Weight, kg | 68.08 ± 11.39 | 66.98 ± 12.43 | 0.092 |  | 68.01 ± 11.24 | 67.59 ± 11.19 | 0.038 |
| Smoking status, n (%) |  |  | 0.212 |  |  |  | 0.013 |
| Non-smoker | 265 (67.4) | 1526 (62.8) |  |  | 218 (65.1) | 217 (64.8) |  |
| Current smoker | 25 (6.4) | 78 (3.2) |  |  | 19 (5.7) | 20 (6.0) |  |
| Ex-smoker | 3 (0.8) | 20 (0.8) |  |  | 3 (0.9) | 3 (0.9) |  |
| NA | 100 (25.4) | 805 (33.1) |  |  | 95 (28.4) | 95 (28.4) |  |
| Drinking status, n (%) |  |  | 0.132 |  |  |  | 0.059 |
| Non-drinker | 267 (67.9) | 1513 (62.3) |  |  | 219 (65.4) | 219 (65.4) |  |
| Current drinker | 18 (4.6) | 114 (4.7) |  |  | 14 (4.2) | 18 (5.4) |  |
| Ex-drinker | 3 (0.8) | 12 (0.5) |  |  | 3 (0.9) | 3 (0.9) |  |
| NA | 105 (26.7) | 790 (32.5) |  |  | 99 (29.6) | 95 (28.4) |  |
| Family history of CRC, n (%) | 3 (0.8) | 21 (0.9) | 0.011 |  | 3 (0.9) | 3 (0.9) | <0.001 |
| ALB, g/L | 41.86 ± 4.24 | 44.26 ± 3.66 | 0.605 |  | 42.47 ± 3.91 | 42.20 ± 4.00 | 0.07 |
| ALT, U/L | 14.0 (10.5, 18.0) | 17.0 (13.0, 26.0) | 0.358 |  | 14.0 (11.0, 19.8) | 15.0 (11.2, 19.0) | 0.043 |
| β2-MG, mg/L | 2.24 ± 1.30 | 1.59 ± 0.81 | 0.604 |  | 2.02 ± 0.62 | 1.95 ± 0.88 | 0.093 |
| TC, mmol/L | 4.79 ± 1.02 | 4.96 ± 1.03 | 0.165 |  | 4.87 ± 0.99 | 4.86 ± 1.06 | 0.003 |
| HDL, mmol/L | 1.26 ± 0.31 | 1.36 ± 0.31 | 0.341 |  | 1.28 ± 0.31 | 1.30 ± 0.30 | 0.053 |
| Hypertension, n (%) | 151 (38.4) | 579 (23.8) | 0.319 |  | 121 (36.1) | 107 (31.9) | 0.088 |
| DM, n (%) | 71 (18.1) | 250 (10.3) | 0.224 |  | 60 (17.9) | 58 (17.3) | 0.016 |

Data are presented mean ± SD, median (quartile 1–quartile 3), or N (%).

Abbreviations: SMD, standardized mean difference; ALB, albumin; ALT, alanine aminotransferase; β2-MG, β2-microglobulin; TC, total cholesterol; HDL, high-density lipoprotein; DM, diabetes mellitus; NA, not recorded; CRC, colorectal cancer.
